# Profiling of MicroRNAs for the Identification of Unique and Common MicroRNAs in Preeclamptic Patients of South India Using Next-Generation Sequencing

**DOI:** 10.7759/cureus.70730

**Published:** 2024-10-02

**Authors:** Vinaya Vijayan, Kannan Rajendran, Aparajita D'souza, Y. Subhashini, S. Tarakeswari, B. Ram Reddy, Satish Vemuri

**Affiliations:** 1 Department of Physiology, Saveetha Institute of Medical And Technical Sciences (SIMATS), Tiruvallur, IND; 2 Department of Internal Medicine, Saveetha Medical College and Research Centre, Kancheepuram, IND; 3 Department of Obstetrics and Gynaecology, Employee's State Insurance Corporation (ESIC) Medical College, Hyderabad, IND; 4 Department of Obstetrics and Gynaecology, Fernandez Hospitals, Hyderabad, IND; 5 Department of Physiology, Apollo Institute of Medical Sciences and Research, Hyderabad, IND; 6 Sunshine Medical Academy for Research and Training (SMART) Laboratory, Sunshine Hospital, Hyderabad, IND

**Keywords:** bioinformatics, blood, mirna, placenta, preeclampsia, sequencing

## Abstract

Introduction: Preeclampsia (PE) is a serious pregnancy complication with an unclear cause. Recent studies suggest that microRNAs (miRNAs), particularly miR-1, may play a role in controlling the genes associated with this condition. This study aimed to compare the expression of miRNAs in the blood and placental tissues of women with PE to those with normal pregnancies.

Methods: We conducted small RNA sequencing on blood and placental samples from three groups: (a) early-onset preeclampsia (EOPE), (b) late-onset preeclampsia (LOPE), and (c) normal pregnancies. Bioinformatics tools were used to compare the miRNA profiles across these groups. A total of 744 miRNAs were detected in placental samples, while 913 miRNAs were found in blood samples. We further analyzed the target genes using protein-protein interaction (PPI) maps to understand how these miRNAs may influence gene functions.

Results: Our analysis revealed significant differences in miRNA expression between the EOPE, LOPE, and control groups. Eight miRNAs were consistently detected in both blood and placental samples across all groups, while other miRNAs were either specific to PE or certain tissue types. The 492 target genes identified formed dense interaction networks, with several key genes occupying central roles.

Conclusion: These findings suggest that altered miRNA expression and the resulting disruption of gene networks may contribute to the development of PE. The distinct differences between EOPE and LOPE indicate that these two subtypes may be driven by different underlying mechanisms. This paves the way for future research to explore new treatments targeting these miRNAs and their associated genes.

## Introduction

Preeclampsia (PE) is characterized by hypertension and either proteinuria or other signs of end-organ dysfunction, often involving the liver or kidneys, which develop after 20 weeks of gestation in a previously normotensive woman [1]. Affecting 2%-8% of pregnancies worldwide [2], it remains a major cause of maternal and fetal morbidity and mortality [3]. Although the exact cause of PE is still unclear, it is thought to result from a failure in placental adaptation during the first trimester, leading to systemic maternal inflammation in the second trimester [4]. Despite this, the precise molecular mechanisms behind PE as a hypertensive disorder of pregnancy remain poorly understood. MicroRNAs (miRNAs) are small RNA molecules, approximately 22 nucleotides long, that regulate gene expression post transcription [5]. By binding to complementary sequences in the 3' untranslated regions (3' UTRs) of target messenger RNAs (mRNAs), miRNAs repress translation and promote mRNA degradation [6]. MiRNAs control key cellular processes through these mechanisms, including proliferation, differentiation, apoptosis, metabolism, immunity, and inflammation [7]. Several circulating miRNAs have been implicated in the molecular pathways involved in PE.

Because miRNAs are stable tissue-specific molecules that can be detected in bodily fluids, they have been suggested as potential diagnostic biomarkers for various pregnancy complications [8, 9]. Studies examining preeclamptic patients' placental, plasma, serum, and urine samples have revealed altered miRNA expression profiles compared to healthy pregnancies [10]. Factors such as geographic origin, ethnicity, genetic background, and environmental influences are known to impact miRNA expression patterns [11]. However, most of the research on miRNA expression changes in PE has been conducted in Western populations, with only limited studies focusing on South Indian patients.

Therefore, it is crucial to conduct further studies to elucidate the role of miRNAs in the development of PE among South Indian women. Next-generation sequencing (NGS) offers powerful tools for differential miRNA transcriptome quantification and the identification of novel miRNA species, thanks to its high throughput capabilities [12]. The primary objective of this study is to identify novel, and potentially shared, dysregulated miRNA biomarkers in South Indian preeclamptic patients using NGS analysis. These differentially expressed miRNAs could serve as candidate biomarkers for the early diagnosis and prognosis of PE. Furthermore, this research may provide insights into the molecular mechanisms underlying the disease in this underexplored ethnic population.

The aim of this research is to analyze the role played by microRNAs in the development of PE, by comparing their expression in the blood and placental tissues of women with early-onset preeclampsia (EOPE), late-onset preeclampsia (LOPE), as well as normal pregnancies. The study focuses on identifying different expressions of miRNAs in preeclamptic and normal pregnancies, analyzing their target genes using bioinformatics tools, and exploring the molecular mechanisms that underlie PE, particularly among South Indian women. This exploration aims to identify probable diagnostic biomarkers and therapeutic targets to improve early diagnosis and treatment of PE.

## Materials and methods

The Employees State Insurance Corporation (ESIC) Medical College, Saveetha Institute of Medical and Technical Sciences, Fernandez Hospital in Hyderabad, the Department of Molecular Genetics at Redcliffe Laboratories, and other tertiary care hospitals collaborated to conduct this multicentric study in the Department of Obstetrics and Gynecology. This study took two years and three months, from January 2020 to March 2022.

Information on ethical approvals

The relevant institutes' institutional ethics committee (IEC) approved the investigation and issued IEC license numbers (008/09/2019/IEC/SMCH, ESICMC/SNR/IEC-S101/12-2020 (ESIC), and No. 32 2020 (Fernandez Hospital)). The participants in the study gave their informed, written consent.

We ensured the study complied with all applicable international ethical standards by the Helsinki Declaration's Ethical Principles for Medical Research Involving Human Subjects (World Medical Association, 1964).

Inclusion criteria

The control group selected seven healthy pregnant women who were at least 34 weeks pregnant. The study included three patients with EOPE and three patients with LOPE. The American College of Obstetricians and Gynecologists (ACOG) guidelines (2019) were referenced when defining PE [13].

Exclusion criteria

Pregnant women with co-occurring conditions or health problems like persistent hypertension, autoimmune diseases, or gestational diabetes were excluded. Any women who were currently taking any other medications were not included in the research, and a detailed clinical history and a pharmacological history were obtained using a defined proforma.

Methods

Blood samples were obtained at the time of birth and placed in a unique microRNA Pax tube, which was kept at -200°C for further analysis. Placental samples (1 cm thick near the chorionic plate) were obtained at the time of transfer and stored in RNA later under -200 °C until processing.

MicroRNA Extraction

MicroRNAs were extracted using the miRNeasy Micro Kit (Qiagen, Venlo, The Netherlands) by following the manufacturer’s protocol. The extracted RNA was quantitatively and qualitatively checked using a Qubit 4.0 fluorometer (Thermo Fischer Scientific, Waltham, MA) and TapeStation 4150 (Agilent Technologies, Inc., Santa Clara, CA). All solutions were prepared using RNase-free, DNase-free ultrapure water.

Small RNA Library Preparation and Sequencing

MicroRNA libraries were made using the MGIEasy Small RNA Library Prep Kit (MGI Tech Co., Ltd., Shenzhen, China) on MGISP100 (MGI Tech Co., Ltd.), which is an automated library preparation system. The library profiles were checked using Tapestation 4150. The libraries were quantified using a Qubit 4. 0 fluorometer (Agilent Technologies, Inc.), and sequencing was performed on the DNBSEQ-G400 sequencer (MGI Tech Co., Ltd.) with an average of 30 million reads per sample.

Bioinformatics analysis

Data QC, Known and Novel miRNA Prediction

The quality of raw miRNA sequencing reads of 50 bp read length was checked through the Fast Quality Control (QC) toolkit (Andrews SC, 2010, http://www.bioinformatics.babraham.ac.uk/projects/fastqc), followed by the removal of low-quality reads and the adapter from the sequences using the Cutadapt program (Marcel Martin, Berlin, Germany). The filtered reads were processed further for the estimation of total and unique reads of 15 to 35 nt reads in each sample individually, which were later mapped on the human reference genome (hg19) using the mapper module of the mirDeep2 algorithm (developed by Marc Friedländer based at the Max Delbrück Center for Molecular Medicine (MDC), Berlin, Germany). Human-based known or conserved miRNAs were identified by miRbase database version 22.1 (developed by Sam Griffiths-Jones and his team at the Faculty of Biology, Medicine and Health, University of Manchester, Manchester, United Kingdom) [14]. Similarly, novel miRNAs were predicted through the mirDeep2 algorithm, which was manually curated for the significant Randfold P-value.

Differential expression estimation

The miRNAs having log2fc > 1 were denoted as upregulated miRNAs, while log2fc < 1 was represented as downregulated miRNAs. Some exclusively expressed miRNAs were also present, which is specific to the particular conditions.

Target prediction and annotation

The upregulated, downregulated, and exclusively expressed miRNAs were used as input for the target prediction in the mirDIP database. mirDIP is a miRNA data integration portal by which targets for miRNAs were predicted in homo sapiens, which was annotated further through the Uniport database [15]. To find the comprehensive study of miRNAs with PE, their respective 913 unique genes were downloaded from the National Center for Biotechnology Information (NCBI) database by using the keywords “preeclampsia.” The identified differentially expressed miRNAs were mapped against the PE-related genes and counted their frequency.

Network analysis and identification of key genes 

The network analysis was carried out using the genes specifically involved in preeclampsia in all the differential miRNA expression comparisons. The network analysis was carried out using Cytoscape, version 3.9.1 (Cytoscape Consortium, 2016) [16], followed by the hub gene identification using the cytoHubba plugin (developed by Chin-Hsien (Hubert) Wu and colleagues at Chang Gung University, Taoyuan, Taiwan) [17].

## Results

Raw data statistics

**Figure 1:**
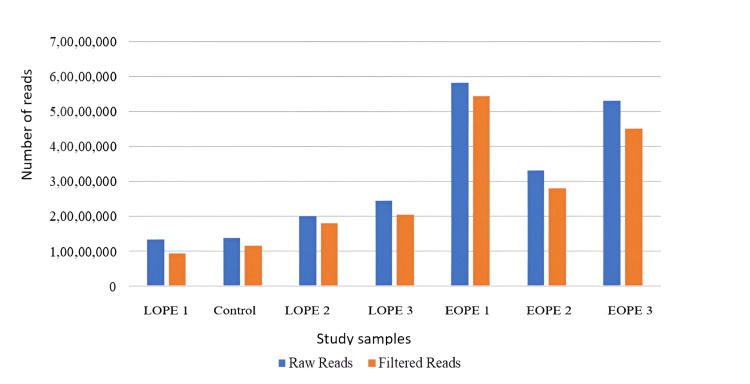
Raw and adaptor-filtered reads in tissue samples; the X-axis is the sample, while the Y-axis shows the number of reads. LPE: late-onset preeclampsia; EOPE: early-onset preeclampsia

**Figure 2:**
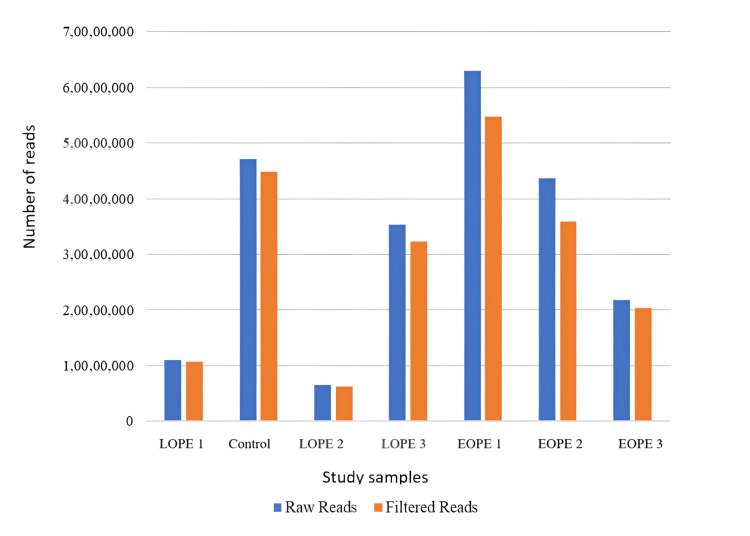
Read statistics of raw and adaptor-filtered reads in blood samples; the X-axis represents the samples, and the Y-axis displays the read counts. LPE: late-onset preeclampsia; EOPE: early-onset preeclampsia

Raw and filtered read statistics of each sample in blood and tissue samples were estimated, in which 82% to 97% of reads comprised high-quality reads in blood samples, while 70% to 93% represented the high-quality reads in tissue samples (Figures 1, 2).

Pre-filtering the raw reads yielded high quantities of filtered reads exclusively in all the subjects in tissue and blood specimens. The tissue samples in Figure 1, collected from five different individuals, had higher raw read counts than the filtered ones, though the difference was not as significant as observed in the other four samples. In Figure 2, with the blood samples, raw reads surpassed filtered ones for all the individuals to a greater or lesser degree. These patterns were obtained for individuals across the conditions, including LOPE 1, control, LOPE 2, LOPE 3, EOPE 1, EOPE 2, and EOPE 3. A certain fluctuation was identified, but raw reads were always higher than filtered reads within the same condition in one or the other individuals. For example, while comparing the raw reads obtained from tissue and blood samples of one individual, the raw reads in all the tested conditions were significantly higher. Taken together with these results, the raw reads-to-filtered read ratios were found to be systematically higher in both sample types across individuals and conditions taken together, conveying a general picture regarding the laid relationship between raw and filtered reads in the existing datasets examined under the present study.

Length distribution graph

**Figure 3:**
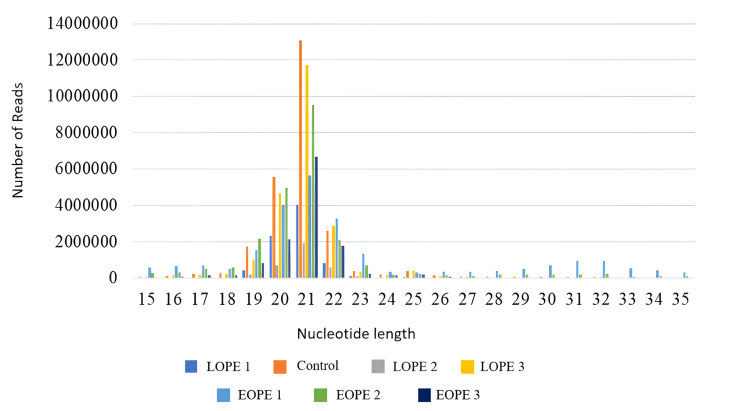
Read distribution of filtered reads in blood samples; the X-axis shows the nucleotide length, and the Y-axis shows the number of reads. LPE: late-onset preeclampsia; EOPE: early-onset preeclampsia

**Figure 4:**
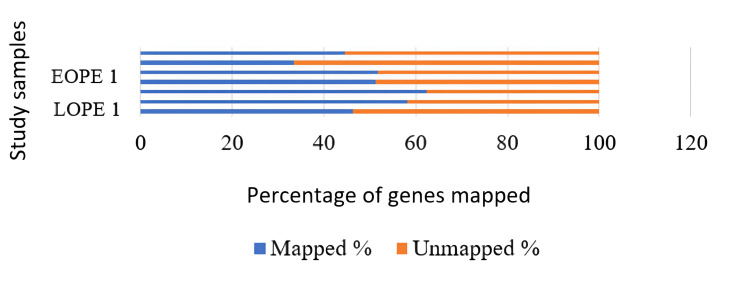
The percentage of miRNAs that could be mapped to a genome in tissue samples; the genes could be mapped from only two tissue samples. LPE: late-onset preeclampsia; EOPE: early-onset preeclampsia; miRNA: microRNAs

The distribution of uniquely filtered reads of 15 to 35 nt length of each sample in blood and tissue samples has been estimated, in which almost all samples had a higher abundance of 20-22 nt reads, which supports the accuracy of miRNA sequencing, and we see a bell-shaped length distribution curve in Figures 3, 4.

A clear distinction in the data distribution between the chromosome and tissue and blood samples was observed across the different experimental conditions. The data were concentrated most significantly in chromosomes 20 and 21 among all analyzed samples of schizophrenic patients as well as in the control group. In these regions, the highest count values were obtained for LOPE 1 and EOPE 1 in tissue; the highest count values reached 375 and 425, respectively; for blood, the highest count value was reached under both LOPE 3 and EOPE 1, which are 300 each. Tissue samples also had a distinct hump on chromosome 32 for the EOPE 2 condition, which registered a count of 225. The remaining chromosomes therefore showed generally smooth, though slightly less intensive, profiles in both sample types.

Genome mapping

**Figure 5:**
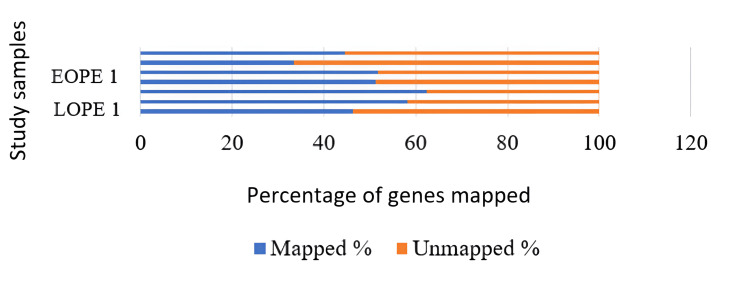
The percentage of miRNAs that could be mapped to a genome in tissue samples; the genes could be mapped from only two tissue samples. LPE: late-onset preeclampsia; EOPE: early-onset preeclampsia; miRNA: microRNAs

**Figure 6:**
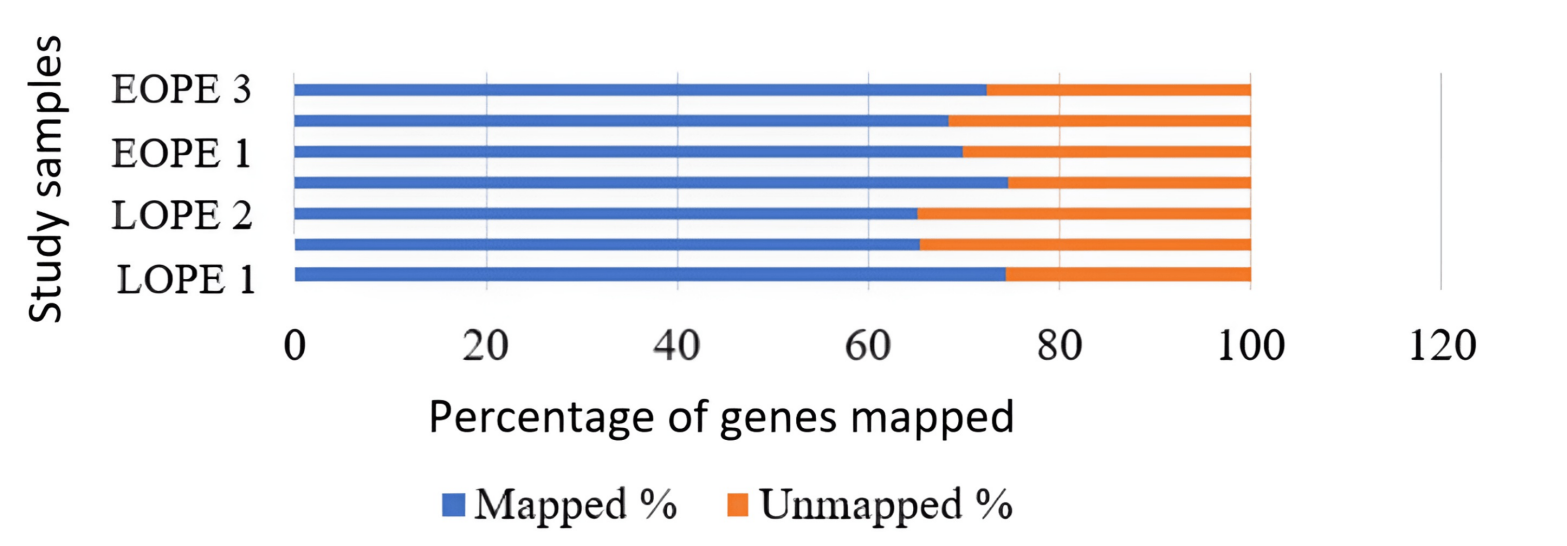
The percentage of miRNAs that could be mapped to a genome in blood samples; the genes could be mapped from two EOPE and two LOPE samples. LPE: late-onset preeclampsia; EOPE: early-onset preeclampsia; miRNA: microRNAs

Distinct filter reads were used to align the data collected to the human reference genome bowtie1 aligner that was in-built into the mapper module of the mirDeep2 algorithm. A total of 65% to 74% of unique reads were mapped on the reference genome for the blood samples, in which the LOPE-2 sample and LOPE-1 had the lowest and highest mapping reads, respectively. Likewise, in tissue samples, EOPE-2 has the lowest mapping percentage, followed by EOPE-1, LOPE-1, LOPE-3, EOPE-3, control, and LOPE-2. The LOPE-2 had the highest mapping percentage reads (62.47%) in the tissue sample (Figures 5, 6).

Comparative miRNA analysis between LOPE, EOPE, and control samples

Around 744 microRNAs were found to be conserved in the tissue samples. Out of these 416 miRNAs, they were found to be common in all the samples for LOPE, EOPE, and control. Forty-five miRNAs were found exclusively in controls; 59 miRNAs were found exclusively in EOPE samples, and 84 microRNAs were found exclusively in LOPE samples. Fifty-five microRNAs were expressed in common between control and LOPE. Thirty microRNAs were expressed in common between control and EOPE. Fifty-five microRNAs were expressed in between LOPE and EOPE.

**Figure 7:**
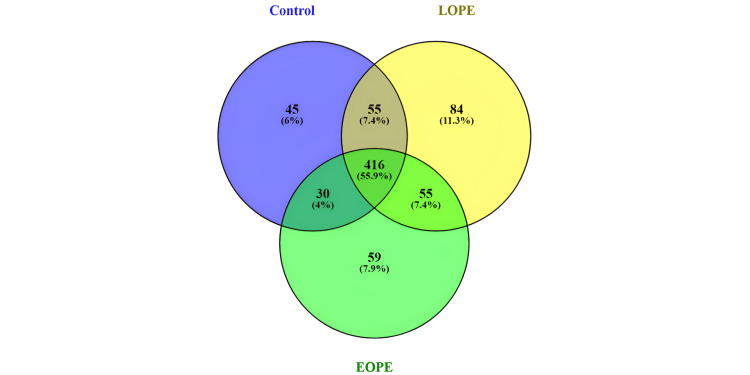
A Venn diagram of known miRNA in tissue samples LPE: late-onset preeclampsia; EOPE: early-onset preeclampsia; miRNA: microRNAs

**Figure 8:**
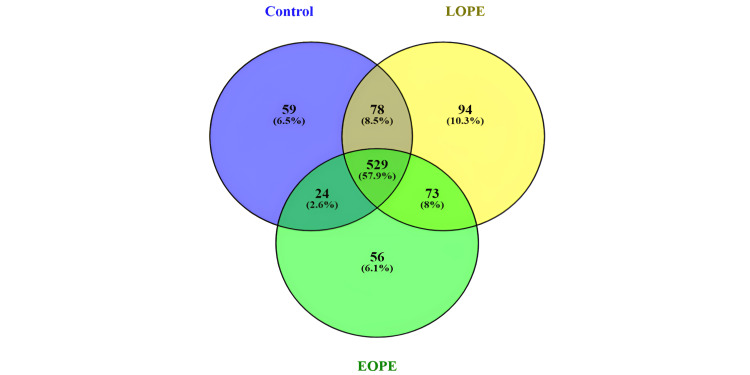
A Venn diagram of miRNAs between control, LOPE, and EOPE in blood LPE: late-onset preeclampsia; EOPE: early-onset preeclampsia; miRNA: microRNAs

A total of 913 microRNAs were expressed in blood samples, out of which 529 miRNAs were found commonly in three groups, i.e., control, LOPE, and EOPE. Fifty-nine microRNAs were found exclusively in control samples. Fifty-six microRNAs were found exclusively in EOPE samples, and 94 microRNAs were found exclusively in LOPE samples. Seventy-eight microRNAs were found to be expressed in common between control and LOPE. Twenty-four microRNAs were expressed in common between control and EOPE, and 73 microRNAs were expressed between LOPE and EOPE. Venn diagrams for the comparative miRNAs in control, LOPE, and EOPE for both blood and tissue samples are depicted in Figures 7, 8.

Network analysis and hub genes

**Figure 9:**
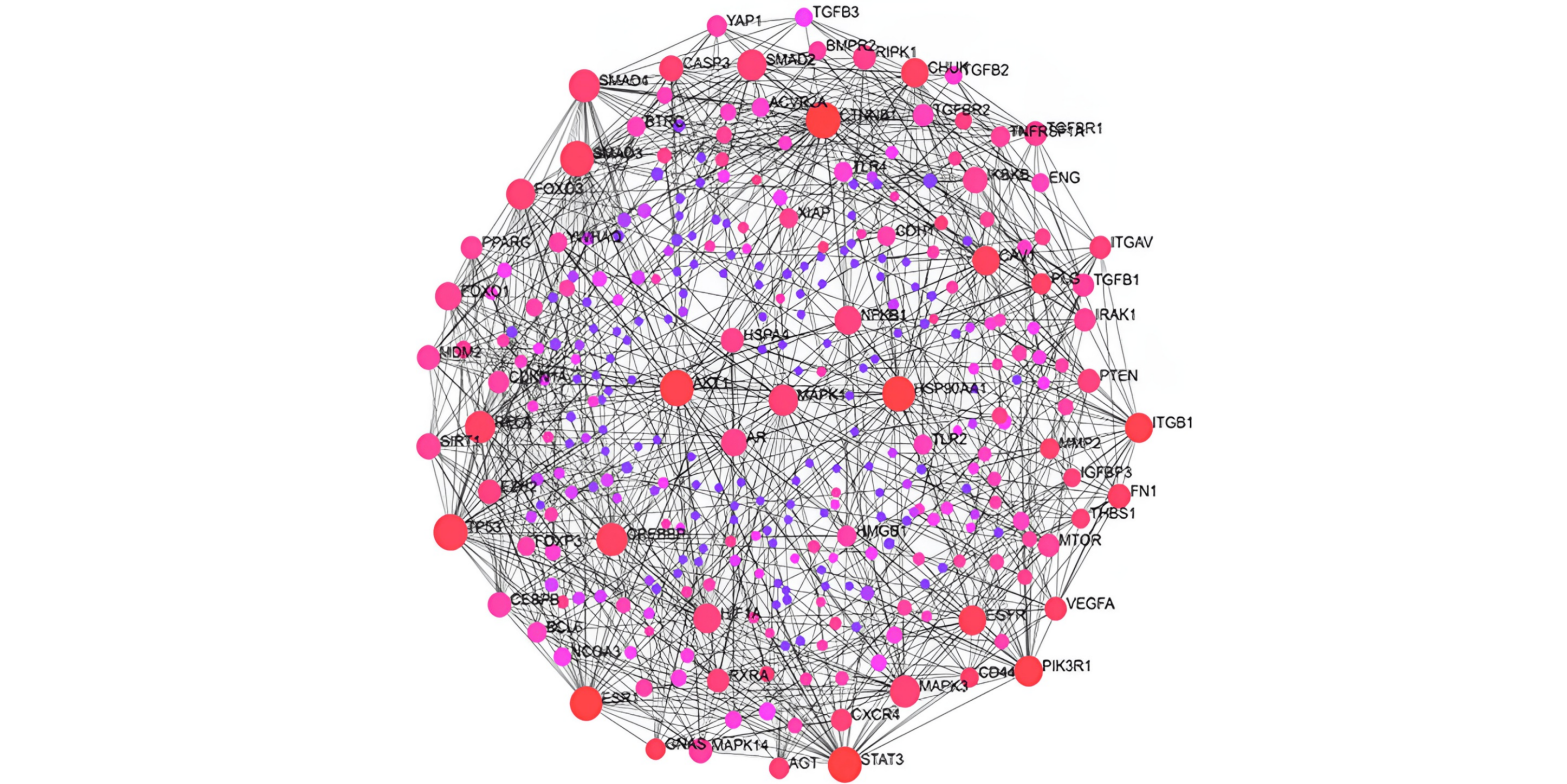
Zero-order protein-protein interaction (PPI) network related to preeclampsia genes This figure was created by using software tools STRING for PPI network creation [18], Cytoscape for network visualization [16], and the cytoHubba plugin to identify hub genes [17].

The target genes that were specifically mined for PE concerning the up- and down-regulated miRNAs were taken together, and the total number of PE genes was found to be 492. These genes were taken up for the protein-protein interaction (PPI) network analysis using the STRING Interactome database with a confidence score cutoff of 900 [18]. A zero-order network was constructed with 325 nodes, 1,010 edges, and 325 seeds. Network topology in terms of degree distribution is depicted below, which had a clustering coefficient of 0.23. The zero-order PPI network of the genes is depicted in Figure 9.

The hub genes were mined from the complete PPI network using the cytoHubba plugin from Cystoscope. This resulted in the identification of 10 potential hub genes based on the degree, to which can be major players in PE. The hub gene network is depicted in Figure 9. The top 10 HUB genes include CTNNB1, TP53, AKT1, STAT3, SMAD3, HSP90AA1, ESR1, SMAD4, CREBBP, and MAPK3.

## Discussion

Preeclampsia is a pregnancy-associated condition of multiple organ dysfunction of unknown cause. Some of the causes include inadequate formation of the placenta due to inadequate invasion of trophoblasts and occlusion of the spiral arteries. These factors can lead to intrauterine growth restriction, an inflammatory hypoxic environment, and an imbalance of angiogenic and anti-angiogenic [11]. Preeclampsia impacts 2%-5% of pregnancies worldwide [12]. African American, Hispanic, and White subgroups have all been compared in research conducted in the United States to evaluate the relationship between ethnicity and severity [19,20]. In India, the prevalence rate is between 8% and 10% [21]. Preeclampsia severity differences between racial and ethnic groups can be attributed to a variety of factors, such as lifestyle choices, social standing, cultural norms, access to care, and utilization of that care [22]. A community-level intervention study conducted in Karnataka identified that maternal health is a construct of several determinants, such as low women's autonomy, inadequate mobility, financial limitations, and incentive-based programs. The destitute socioeconomic profile is a restriction, as women from scheduled castes, scheduled tribes, and the poor presumably had worse health outcomes [23].

The Doppler technique is frequently used in clinical settings to diagnose PE because it indicates a low pulsatile index in preeclamptic cases [24]. The assessment of the condition also makes use of the protein concentration of the urine. Recently, biochemical markers including soluble endoglin and sFlt have also been added to illness-detection approaches [25]. Several epidemiological studies have demonstrated that women who have a previous history of PE are at a higher rate of growing chronic hypertension, cardiovascular disease (CVD), and chronic kidney disease. Preeclampsia has been recently classified as a new secondary CVD risk factor for women by the American Heart Association [26]. Preventing related problems with early PE prediction may enhance the monitoring and prognosis of hypertension in pregnant women. Several researchers have focused on finding specific biomarkers for early diagnosis of PE, such as miRNAs [27].

Many distinct miRNAs have been found in placentas associated with PE, and numerous in vivo and in vitro investigations have revealed that these aberrantly expressed miRNAs are a contributing factor to the abnormalities associated with the placenta in PE [28]. Many investigations have focused on the search for specific biomarkers that would help to determine the development of PE in advance, including the main difficulty arising from the fact that the primary pathophysiological changes in PE develop before the 20^th^ week of pregnancy, while clinical symptoms appear only later. Thus, it is important to understand if these placental miRNAs are involved in the pathophysiology of PE or are just products of the disease during the later stages of pregnancy miRNAs [29]. As previously mentioned, pregnant women may release placental miRNAs into the maternal circulation at any time. Differential miRNAs in maternal blood are identified in the placenta.

The present demonstration showed the expression of 604 microRNAs in placental tissue samples. Out of these 416 miRNAs found in common in all the samples, 45 miRNAs were found exclusively in controls, 59 miRNAs were found exclusively in EOPE samples, and 84 microRNAs were found exclusively in LOPE samples. The blood samples showed a total expression of 913 microRNAs, of which 529 microRNAs were expressed commonly in three groups. Fifty-nine microRNAs were found exclusively in control samples. Fifty-six microRNAs were found exclusively in EOPE samples, and 94 microRNAs were found exclusively in LOPE samples [30].

The hub genes that have been associated with PE are CTNNB1, TP53, AKT1, STAT3, SMAD3, HSP90AA1, ESR1, SMAD4, CREBBP, and MAPK3. The CTNNB1 gene is responsible for the beta-catenin protein. This protein is mainly found at the adherents’ junctions, which are complexes that bind two neighboring cells. Beta-catenin is also involved in signaling since it is a component of the Wnt signaling pathway. In this pathway, some proteins that beta-catenin interacts with will help it to get into the nucleus of the cell in these processes. Within the nucleus, beta-catenin associates with other proteins to form a transcription factor that controls the synthesis of some genes. The Wnt signaling pathway is a protein that is involved in the processes of cell division, differentiation, and other developmental processes during fetal development. It is needed in adult tissues for the sustenance of stem cells that can be used in the repair of tissues and also for the stem cells that can develop into any tissue [31].

Another gene linked with PE is HSP90AA1. This gene encodes Hsp90α, which is induced by stress in the cell. Hsp90α is also tissue-specific and is overexpressed in various types of cancer. Another pathway that is pointed out in the development of PE is the MAPK signaling pathway. STAT3 of transcription 3 is a protein that plays a role in the regulation of several significant cellular functions. The immunohistochemical results also confirmed the previous observation that the level of IL-6, surviving, and MMP-2 was down-regulated in the severe preeclamptic placenta as compared to normal placenta. These observations indicate that the downregulation and activation of Stat3 may be a result of the downregulation of an upstream gene such as IL-6. STAT3 and its down-regulation may be very significant in the development of PE through STAT3-targeted genes such as surviving and MMP-2, which are involved in apoptosis and the invasive ability of placental trophoblastic cells [32].

The study observed that the levels of SMAD3 and SMAD4 were lower in EOPE and LOPE than those in the control group of similar gestational age. Smad2 and Smad3 are the R-Smads that are triggered by TGF-β, activin, and nodal type I receptors phosphorylate R-Smads, which then combine with Smad4 to form complexes and go into the nucleus to control the expression of certain genes. In particular, Smad3 stimulates the development of endothelial-like networks, trophoblast invasion, extravillous trophoblast (EVT) sprouting, and genes critical to EVT function [33].

Limitations of the study

The study offers critical insights into microRNA expression differences associated with PE, but a number of limitations should be acknowledged, including a relatively small sample size that could limit the applicability of the findings and a cross-sectional design that prevents causal inferences. Furthermore, analyses did not extensively include some of the factors such as maternal age, BMI, and comorbid conditions, which could affect microRNA expression. As a means to strengthen these discoveries, future research should concentrate on enlarging the sample size to comprise a variety of populations, carrying out longitudinal studies to verify causal relationships, and researching particular microRNAs as prospective biomarkers for early diagnosis, while also considering additional clinical variables to boost early detection and management strategies for PE.

## Conclusions

This study showed considerable differences in microRNA expression levels in blood and placental tissue samples between cases of PE and typical pregnancies, underscoring the potential of these molecules as markers for early detection of PE. Notably, multiple microRNAs were specifically expressed in the samples from preeclamptic patients, implying their central contribution to the pathology of this condition. The lack of extensive investigation into the effect of microRNAs on PE among South Indian women makes this study highly relevant and sets a basis for upcoming studies. The implementation of NGS in this investigation reveals its role in elucidating intricate molecular processes, although it remains limited in access at many labs throughout the nation. The conclusions derived from this study look at the molecular composition of PE and open possibilities for doing more extensive studies to affirm the discovered microRNAs and evaluate their potential applications in clinical settings. Eventually, this work could lead to the introduction of new diagnostic methods and treatment techniques meant to ease the risks of PE, resulting in improved health outcomes for both mothers and their babies in vulnerable communities.

## References

[1] Braunthal S, Brateanu A (2019). Hypertension in pregnancy: pathophysiology and treatment. SAGE Open Med.

[2] Fox R, Kitt J, Leeson P, Aye CY, Lewandowski AJ (2019). Preeclampsia: risk factors, diagnosis, management, and the cardiovascular impact on the offspring. J Clin Med.

[3] Ghulmiyyah L, Sibai B (2012). Maternal mortality from preeclampsia/eclampsia. Semin Perinatol.

[4] Redman CW, Sargent IL (2005). Latest advances in understanding preeclampsia. Science.

[5] Bartel DP (2004). MicroRNAs: genomics, biogenesis, mechanism, and function. Cell.

[6] Jonas S, Izaurralde E (2015). Towards a molecular understanding of microRNA-mediated gene silencing. Nat Rev Genet.

[7] Treiber T, Treiber N, Meister G (2019). Regulation of microRNA biogenesis and its crosstalk with other cellular pathways. Nat Rev Mol Cell Biol.

[8] Hromadnikova I, Kotlabova K, Hympanova L, Krofta L (2016). Gestational hypertension, preeclampsia and intrauterine growth restriction induce dysregulation of cardiovascular and cerebrovascular disease associated microRNAs in maternal whole peripheral blood. Thromb Res.

[9] Kolkova Z, Holubekova V, Grendar M (2021). Association of circulating miRNA expression with preeclampsia, its onset, and severity. Diagnostics (Basel).

[10] Zhu XM, Han T, Sargent IL, Yin GW, Yao YQ (2009). Differential expression profile of microRNAs in human placentas from preeclamptic pregnancies vs normal pregnancies. Am J Obstet Gynecol.

[11] Martinez-Fierro ML, Garza-Veloz I (2021). Analysis of circulating microRNA signatures and preeclampsia development. Cells.

[12] Yang Q, Lu J, Wang S, Li H, Ge Q, Lu Z (2011). Application of next-generation sequencing technology to profile the circulating microRNAs in the serum of preeclampsia versus normal pregnant women. Clin Chim Acta.

[13] American College of Obstetricians and Gynecologists (2019). ACOG Publications: January 2019. Obstet Gynecol.

[14] Kozomara A, Birgaoanu M, Griffiths-Jones S (2019). miRBase: from microRNA sequences to function. Nucleic Acids Res.

[15] (2024). Mirdip: MicroRNA data integration portal. https://ophid.utoronto.ca/mirDIP/.

[16] Shannon P, Markiel A, Ozier O (2003). Cytoscape: a software environment for integrated models of biomolecular interaction networks. Genome Res.

[17] Chin CH, Chen SH, Wu HH, Ho CW, Ko MT, Lin CY (2014). cytoHubba: identifying hub objects and sub-networks from complex interactome. BMC Syst Biol.

[18] Szklarczyk D, Kirsch R, Koutrouli M (2023). The STRING database in 2023: protein-protein association networks and functional enrichment analyses for any sequenced genome of interest. Nucleic Acids Res.

[19] Chen DB, Wang W (2013). Human placental microRNAs and preeclampsia. Biol Reprod.

[20] Jairajpuri DS, Malalla ZH, Sarray S, Mahmood N (2021). Analysis of differential expression of hypoxia-inducible microRNA-210 gene targets in mild and severe preeclamptic patients. Noncoding RNA Res.

[21] GBD 2016 Disease and Injury Incidence and Prevalence Collaborators (2017). Global, regional, and national incidence, prevalence, and years lived with disability for 328 diseases and injuries for 195 countries, 1990-2016: a systematic analysis for the Global Burden of Disease Study 2016. Lancet.

[22] Hu Y, Li P, Hao S, Liu L, Zhao J, Hou Y (2009). Differential expression of microRNAs in the placentae of Chinese patients with severe pre-eclampsia. Clin Chem Lab Med.

[23] Barabási AL, Gulbahce N, Loscalzo J (2011). Network medicine: a network-based approach to human disease. Nat Rev Genet.

[24] Allaire AD, Ballenger KA, Wells SR, McMahon MJ, Lessey BA (2000). Placental apoptosis in preeclampsia. Obstet Gynecol.

[25] Wang YP, Walsh SW, Guo JD, Zhang JY (1991). The imbalance between thromboxane and prostacyclin in preeclampsia is associated with an imbalance between lipid peroxides and vitamin E in maternal blood. Am J Obstet Gynecol.

[26] Clemente L, Bird IM (2023). The epidermal growth factor receptor in healthy pregnancy and preeclampsia. J Mol Endocrinol.

[27] Chen CP, Bajoria R, Aplin JD (2002). Decreased vascularization and cell proliferation in placentas of intrauterine growth-restricted fetuses with abnormal umbilical artery flow velocity waveforms. Am J Obstet Gynecol.

[28] Praveen M (2024). Characterizing the West Nile virus's polyprotein from nucleotide sequence to protein structure - computational tools. J Taibah Univ Med Sci.

[29] LaMarca B (2010). The role of immune activation in contributing to vascular dysfunction and the pathophysiology of hypertension during preeclampsia. Minerva Ginecol.

[30] Mayor-Lynn K, Toloubeydokhti T, Cruz AC, Chegini N (2011). Expression profile of microRNAs and mRNAs in human placentas from pregnancies complicated by preeclampsia and preterm labor. Reprod Sci.

[31] Zhang M, Deng X, Jiang Z, Ge Z (2022). Identification of underlying mechanisms and hub gene-miRNA networks of the genomic subgroups in preeclampsia development. Medicine (Baltimore).

[32] Parada-Niño L, Castillo-León LF, Morel A (2022). Preeclampsia, natural history, genes, and miRNAs associated with the syndrome. J Pregnancy.

[33] Brkić J, Dunk C, Shan Y (2020). Differential role of Smad2 and Smad3 in the acquisition of an endovascular trophoblast-like phenotype and preeclampsia. Front Endocrinol (Lausanne).

